# A Nationwide Prospective Clinical Trial on Active Surveillance in Patients With Non-intraabdominal Desmoid-type Fibromatosis: The GRAFITI Trial

**DOI:** 10.1097/SLA.0000000000005415

**Published:** 2022-02-15

**Authors:** Anne-Rose W. Schut, Milea J. M. Timbergen, Danique L. M. van Broekhoven, Thijs van Dalen, Winan J. van Houdt, Johannes J. Bonenkamp, Stefan Sleijfer, Dirk J. Grunhagen, Cornelis Verhoef

**Affiliations:** * Department of Surgical Oncology, Erasmus MC Cancer Institute, Rotterdam, The Netherlands; † Department of Medical Oncology, Erasmus MC Cancer Institute, Rotterdam, The Netherlands; ‡ Department of Orthopaedics and Sports Medicine, Erasmus University Medical Center, Rotterdam, The Netherlands; § Department of Surgical Oncology, University Medical Center Utrecht, the Netherlands; ¶ Department of Surgery, Diakonessenhuis Utrecht, The Netherlands; || Department of Surgical Oncology, Netherlands Cancer Institute, Amsterdam, The Netherlands; ** Department of Surgical Oncology, Radboud University Medical Center, Nijmegen, The Netherlands

**Keywords:** active surveillance, aggressive fibromatosis, desmoid tumor, treatment outcome, wait-and-see, watchful waiting

## Abstract

To assess tumor behavior and the efficacy of active surveillance (AS) in patients with desmoid-type fibromatosis (DTF).

**Summary of Background Data:**

AS is recommended as initial management for DTF patients. Prospective data regarding the results of AS are lacking.

**Methods:**

In this multicenter prospective cohort study (NTR4714), adult patients with non-intraabdominal DTF were followed during an initial AS approach for 3 years. Tumor behavior was evaluated according to Response Evaluation Criteria in Solid Tumors. Cumulative incidence of the start of an active treatment and progression-free survival (PFS) were calculated using the Kaplan-Meier method. Factors predictive for start of active treatment were assessed by Cox regression analyses.

**Results:**

A total of 105 patients started with AS. Median tumor size at baseline was 4.1cm (interquartile range 3.0–6.6). Fifty-seven patients had a T41A CTNNB1 mutation; 14 patients a S45F CTNNB1 mutation. At 3 years, cumulative incidence of the start of active treatment was 30% (95% confidence interval [CI] 21–39) and PFS was 58% (95% CI 49–69). Median time to start active treatment and PFS were not reached at a median follow-up of 33.7 months. During AS, 32% of patients had stable disease, 28% regressed, and 40% demonstrated initial progression. Larger tumor size (≥5 cm; hazard ratio = 2.38 [95% CI 1.15–4.90]) and S45F mutation (hazard ratio = 6.24 [95% CI 1.92–20.30]) were associated with the start of active treatment.

**Conclusions:**

The majority DTF patients undergoing AS do not need an active treatment and experience stable or regressive disease, even after initial progression. Knowledge about the natural behavior of DTF will help to tailor the follow-up schedule to the individual patient.

Desmoid-type fibromatosis (DTF) is a rare soft-tissue tumor with a highly variable clinical course. Adults are mostly affected and tumors can be located at nearly any body site, including the extremities, the abdominal wall, and intraabdominal locations.^1^ The majority of DTF tumors are sporadic and characterized by mutations in exon 3 of the β-catenin (*CTNNB1*) gene, including T41A, S45F, and S45P.^2–4^ In 5% to 10%, DTF arises in the context of familial adenomatous polyposis (FAP), which is associated with mutations in the (adenomatous polyposis coli) *APC* gene.^5,6^ Tumors lacking mutations in the *CTNNB1* or *APC* gene are categorized wild-types.^2–4^ The development of sporadic DTF is not fully understood, but has been related to etiological factors as surgical trauma and hormonal influences.^7,8^ In FAP patients, DTF is mainly located at intraabdominal sites. The association between intraabdominal DTF and FAP is suggestive for a different tumor biology and subsequently a different treatment strategy compared to sporadic disease.^6,9^ DTF cannot metastasize, but can display local infiltrative growth and has a tendency to recur locally after surgery. The biological behavior is unpredictable, exhibiting phases of initial progression, growth stabilization, or frequently even regression without any treatment, which makes DTF challenging to treat.^5,10^ Independent of tumor behavior and size, symptoms can vary between being completely absent to extremely painful and function limiting situations.

Up to 10 years ago, surgery was the mainstay of DTF treatment, but high local recurrence rates and the high numbers of spontaneous regression caused a shift to a more conservative approach.^11–14^ First, an active surveillance (AS) approach was only

offered to patients with recurrent tumors, but in the last years it is considered standard of care in primary DTF as well.^12,14–17^ Currently, the latest guidelines suggest AS as initial management for asymptomatic and mildly symptomatic patients, independent of tumor size and site. In case of persistent radiological or symptomatic progression active treatment with systemic therapy, surgical resection, or radiotherapy may be considered.^18^

Identifying factors predictive for the failure of an AS approach will help physicians and patients to choose the appropriate treatment strategy upfront, leading to a more personalized treatment approach. Several potential clinicopathological factors associated with change in treatment strategy and risk of progression or recurrence have been evaluated in retrospective studies, such as tumor size, tumor location, and *CTNNB1* mutation status. However, drawing a single conclusion remains challenging due to variable treatment regimens and heterogeneous patient cohorts, which emphasizes the need for a prospective evaluation.^13,16,19–21^

The aim of the GRAFITI trial was to prospectively assess tumor behavior of DTF during an AS approach in adult patients with non-intraabdominal DTF. Furthermore, the efficacy of an AS approach as initial management was evaluated, including identification of predictive factors for success or failure of an upfront AS approach.

## Methods

### Study Design and Population

The GRAFITI trial was a prospective, multicenter observational study performed in 7 sarcoma centers in the Netherlands. The study was approved by the Ethics Committee of the Erasmus Medical Centre (MEC-2014-124), registered in the Dutch trial register (study ID: NTR4714) and its design has been published previously.^9^ Patients with non-intraabdominal tumor localization, a histologically proven diagnosis of DTF and without previous treatment for the current lesion were eligible for inclusion. Patients <18 years, with personal or family history of FAP, with severe pain or functional impairment due to the tumor (as indicated by the patient; use of analgesics, including nonsteroidal antiinflammatory drugs [NSAIDs], was not an exclusion criterion) or with tumor progression leading to mutilation or life/limb-threatening situations as assessed by the treating physician were excluded. Inclusion was open from May 2014 until December 2018.

### Study Procedures

Patients with suspected or confirmed DTF referred to one of the participating centers were evaluated for eligibility for inclusion. Reasons for exclusion were documented. Eligible patients who provided written informed consent were included in the study. AS is defined as continuous monitoring of DTF patients with an initial Magnetic Resonance Imaging scan (MRI) (or alternatively another imaging modality when MRI is unavailable) within 1 to 2 months, followed by imaging with intervals according to the European consensus guideline.^18^ The follow-up protocol of the GRAFITI trial consisted of follow-up visits and imaging examinations (Ultrasound [US] and MRI) at baseline, 3,6,9,12,18,24, and 36 months (window ±3 months).^9^ Findings on physical examination, medication, hormonal status (females only), pain score (1–10), and presence of symptoms reported by the treating physician were recorded at each follow-up visit. Symptoms were considered absent when there was no documentation of symptoms and present when the treating physician reported any symptoms. *CTNNB1* mutation status was assessed at baseline on the basis of pathology reports for cases with known *CTNNB1* mutation status or by Sanger Sequencing when *CTNNB1* mutation status was unknown and pathology specimens were available. If biopsy material was unavailable or insufficient for further analysis, the *CTNNB1* mutation status remained unknown. Tumor localization and maximum diameter at baseline and during follow-up were assessed by a radiologist. Tumor behavior of DTF was evaluated according to the Response Evaluation Criteria in Solid Tumors (RECIST) version 1.1 and defined as progressive disease (PD), stable disease (SD), partial regression (PR), or complete regression (CR).^22^ To minimize measurement variability, only MRI-images were used to analyze tumor size and tumor behavior. Measurements from computed tomography or US were only used in case MRI-images were not available and all measurements during follow-up were performed using the same imaging technique.

The decision to start treatment was individually made by both the physician and the patient and was discussed in a multidisciplinary meeting. Reasons for re-evaluating the current AS management strategy were tumor growth or progressive symptoms according to the international guidelines.^18^ When AS was no longer feasible, active treatment was started and tumor behavior according to RECIST and the reason for change in treatment were documented. Symptomatic progression was determined according to the documentation in the electronic patient record and considered present if an increase in symptoms was described by the treating physician as one of the reasons for initiating active treatment. Active treatments included systemic therapy, surgical resection, or radiotherapy according to the European consensus guidelines.^18^ Treatment with NSAIDs or other analgesics was not considered as an active treatment in the current study as there is no evidence for the use of NSAIDs as antitumor therapy in DTF.^18^ The end of follow-up was marked by the start of active treatment or the last registered contact between physician and patient. After3 years of AS, further follow-up was determined by the treating physician and data were collected when available.

### Outcomes

The primary endpoint reported here was progression-free survival (PFS), defined as the time from inclusion to the date of first PD or death from any cause. Secondary endpoints were the cumulative incidence of the start of an active treatment, considerations for active treatment, and factors predictive for failure of AS. The complete list of the endpoints is reported in the previously published protocol.^9^

### Statistical Analysis

Based on the incidence of DTF, enrolment was estimated at 20 patients annually.^11^ A total of 100 patients was expected to be included during a period of maximum 5 years. With a sample size of 100 patients, a progression rate of 50% would result in a 95% confidence interval (CI) of 40% to 60% and a progression rate of 25% would result in a 95% CI of 18% to 34% at a 2-sided significance level of 0.050. These 95% CIs were considered as acceptable for this study.^9^

Continuous variables were presented as median and interquartile range (IQR). Categorical variables were described as numbers and percentages. Comparative analyses were performed with χ^2^ tests for categorical variables and Mann-Whitney *U* tests for continuous variables. The Kaplan-Meier method was used to estimate the cumulative incidence of the start of an active treatment and the PFS, with censoring at the last follow-up for patients who did not start an active treatment or experienced PD respectively. Univariable Cox regression analyses were performed to assess possible factors associated with start of active treatment, and results are presented as hazard ratios (HRs) with 95% CI. Multivariable Cox regression was performed using variables that were statistically significant in univariable analysis.

A planned interim analysis was performed after 1 year of follow-up from the first 20 patients to evaluate the number of patients who needed to switch to an active treatment. The study was considered safe if >50% of the patients were still undergoing AS after 1 year of follow-up. Statistical analyses were performed using SPSS Statistics (IBM, Armonk, NY, version 25.0) and R version 3.6.1. (http://www.r-project.org/). Figures were generated with GraphPad Prism version 5.0 (GraphPad Software, La Jolla, CA). For all analyses, 2-sided *P* < 0.050 was considered statistically significant.

## Results

### Patient Characteristics

A total of 164 patients with suspected or diagnosed DTF were referred to one of the participating centers. Fifty-eight patients were not eligible for study participation, leaving 106 patients who started with an AS approach (Supplemental Digital Content Table 1.

**Table 1:** Baseline Characteristics of Included Desmoid-type Fibromatosis Patients

	(*N* = 105) *n* (%)
Age at time of diagnosis (years)	
Median (IQR)	37 (32–47)
Sex	
Male	21 (20)
Female	84 (80)
Tumor localization	
Abdominal wall	37 (35)
Head and neck	8 (8)
Upper extremity	7 (7)
Trunk and back	25 (24)
Breast	10 (9)
Lower extremity	18 (17)
Recurrent disease	
Yes	6 (6)
No	99 (94)
Tumor size (cm)	
Median (IQr)	4.1 (3.0–6.6)
<5	60 (57)
5–10	38 (36)
>10	7 (7)
*CTNNB1* mutation status^1^	
T41A	57 (54)
S45F	14 (13)
S45P	16 (15)
WT	8 (8)
Others	3 (3)
Unknown	7 (7)
Previous surgery in area of current DTF tumor	
Yes	23 (22)
Hormonal status at time of inclusion^*^	
Premenopausal	69 (82)
Postmenopausal	14 (17)
Pregnant	1 (1)
History of pregnancy before diagnosis of DTF^*^ (*n* = 81)^2^	
Yes	63 (75)
Use of hormonal medication at inclusion (*n* = 104)^2^	
Yes^3^	20 (19)
Use of NSAIDs at inclusion (*n* = 103)^2^	
Yes	5 (5)
Symptoms at time of inclusion^4^	
Yes	68 (65)

^*^Only in female population (*n* = 84).

^1^WT: wild-type; Others: S33L, H36P, Ser33Tyr; Unknown: insufficient/unavailable material to determine *CTNNB1* mutation status.

^2^Number of patients with known pregnancy status or medication use.

^3^All hormonal medication involved hormonal contraceptives.

^4^Sensory symptoms, motoric symptoms, cosmetic complaints, pain, cramps.

The majority of the patients were females (80%) with a median age of 37 years (IQR 32–47) at time of diagnosis. Most common tumor locations were the abdominal wall (35%) and the trunk and back (24%). Median tumor size at baseline was 4.1 cm (range 3.0–6.6). The majority (54%) had a T41A mutation. Five patients (5%) used NSAIDs at the time of inclusion, of whom 3 patients chronically used NSAIDs for another indication and 2 patients used NSAIDs for pain due to their DTF.

### Treatment Strategy During Follow-up

**Figure 1:**
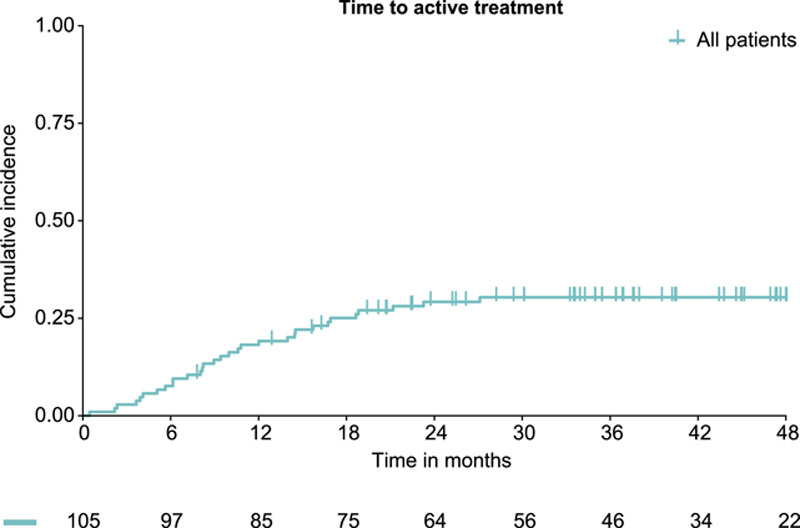
Cumulative incidence of the start of an active treatment in 105 patients initially managed with active surveillance.

The first 20 patients who completed at least 1 year of follow-up were included in the planned interim analysis. Fifteen of 20 patients were still undergoing AS (75%) and the AS approach was considered safe. Of the 105 patients with an initial AS approach, 31 (30%) discontinued AS and started with some form of active treatment during follow-up. Median time to the initiation of active treatment was not reached at a median follow-up of 33.7 months (IQR 15.6–47.0). Overall, the incidences of starting active treatment at 1 and 3 years were 18% (95% CI 10–25) and 30% (95% CI 21–39), respectively (Fig. 1). The remaining 74 patients (70%) continued with AS until their last follow-up, with a median follow-up of 39.1 months (IQR 32.3–49.6). None of the patients who continued AS and with an available follow-up moment switched to active treatment at 3 to 4 (*n* = 34) and 4 to 5 years (*n* = 10) of follow-up.

**Figure 2:**
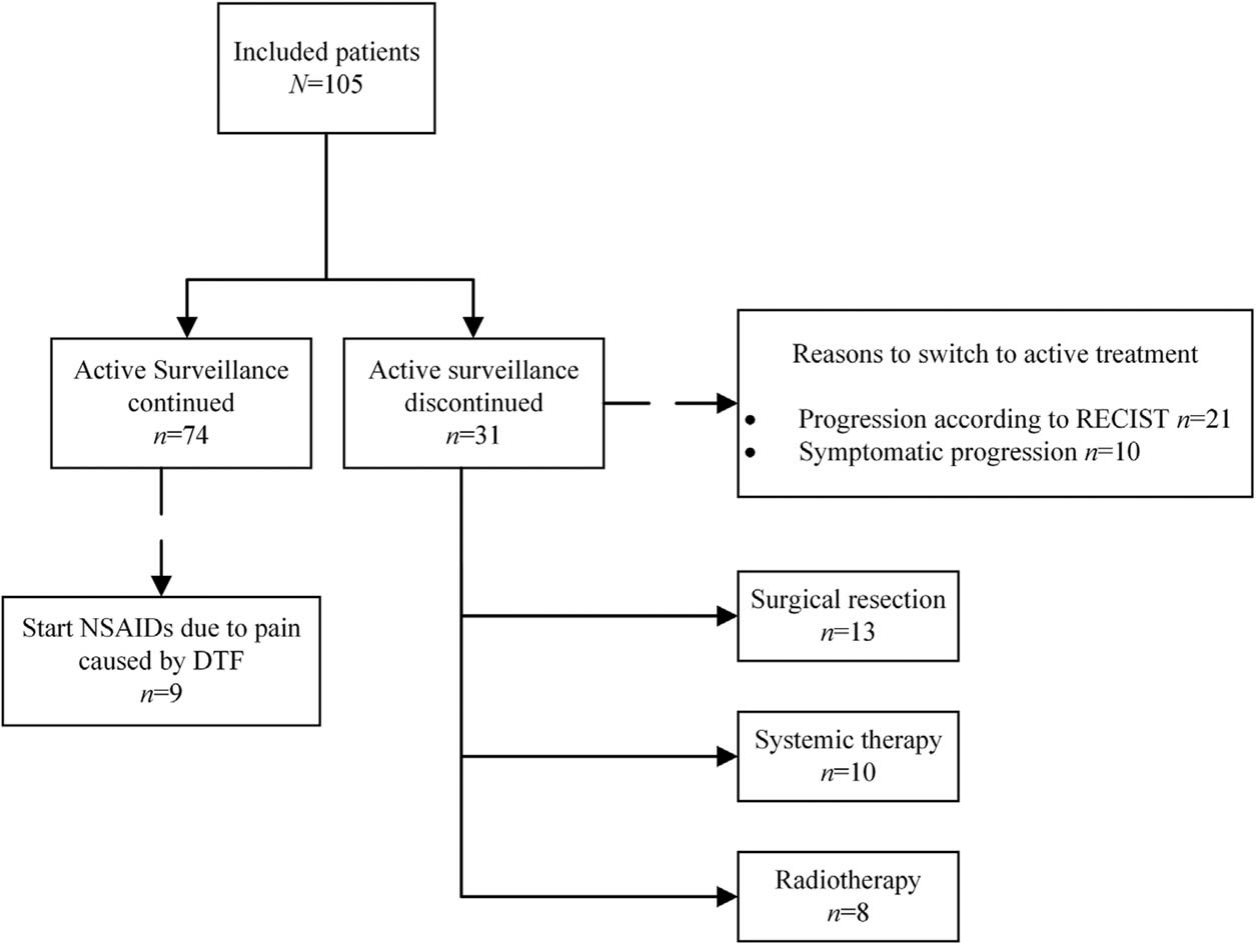
Treatment strategies during follow-up. Systemic therapy included treatment with doxorubicine, vinorelbine, or tamoxifen.

The treatment strategy during follow-up is summarized in Figure 2. Nine patients started with NSAIDs due to pain caused by their DTF and were able to continue AS. Reasons to start active treatment included PD according to RECIST with or without increase in symptoms (*n* = 21) or symptomatic progression (*n* = 10).

Univariable analysis of factors affecting the risk of starting active treatment showed that larger tumor size (≥5cm; HR = 2.38 [95% CI 1.15–4.90]) and the presence of a S45F mutation (HR = 6.24 [95% CI 1.92–20.30]) were associated with a higher risk of starting active treatment (Table 2).

**Table 2:** Univariable and Multivariable Analyses of Factors Influencing the Risk of Starting Active Treatment

	Active Surveillance (*n* = 74)	Switch to Active Treatment (*n* = 31)	Univariable Analysis		Multivariable Analysis	
	*n* (%)	*n* (%)	HR [95% CI]	*P*	HR [95% CI]	*P*
Age at time of inclusion (median)	37.0	36.0	0.99 [0.96–1.02]	0.481		
Sex				0.717		
Male	14 (19%)	7 (23%)	Ref			
Female	60 (81%)	24 (77%)	0.86 [0.37–1.99]			
Tumor size at baseline (cm)				0.019		0.059
<5	48 (65%)	12 (39%)	Ref		Ref	
≥5	26 (35%)	19 (61%)	2.38 [1.15–4.90]		2.13 [0.97–4.68]	
*CTNNB1* mutation status (*n* = 98)^1^						
Other^2^	23 (34%)	4 (13%)	Ref		Ref	
T41A	40 (59%)	17 (57%)	2.39 [0.80–7.10]	0.118	2.37 [0.80–7.04]	0.122
S45F	5 (7%)	9 (30%)	6.24 [1.92–20.3]	0.002	4.64 [1.38–15.8]	0.013

^1^Unknown *CTNNB1* mutation status were not included in univariable and multivariable analysis.

^2^Other: S45P, S33L, H36P, Ser33Tyr, or wild-type (WT) mutations.

Multivariable analysis using tumor size and *CTNNB1* mutation status only identified the presence of a S45F mutation (HR = 4.64 [95% CI 1.38–15.8]) as a predictive factor for the initiation of active treatment (Table 2). The number and corresponding frequencies of treatment strategy during follow-up, tumor behavior and tumor size according to tumor location and *CTNNB1* mutation type are summarized in Supplemental Digital Content Table 1, http://links.lww.com/SLA/D685 and Supplemental Digital Content Table 2, http://links.lww.com/SLA/D686. The association between tumor size and *CTNNB1* mutation was explored by χ^2^ analysis. A significant correlation between the presence of a S45F mutation and a larger tumor size (≥5 cm) was observed (*P* = 0.004), indicating that tumors harboring a S45F mutation were larger compared to tumors harboring other mutations. No significant correlation could be found between *CTNNB1* mutation and recurrence (*P* = 0.708), age (*P* = 0.170), and sex (*P* = 0.482).

### Natural Behavior of DTF Tumors

**Figure 3:**
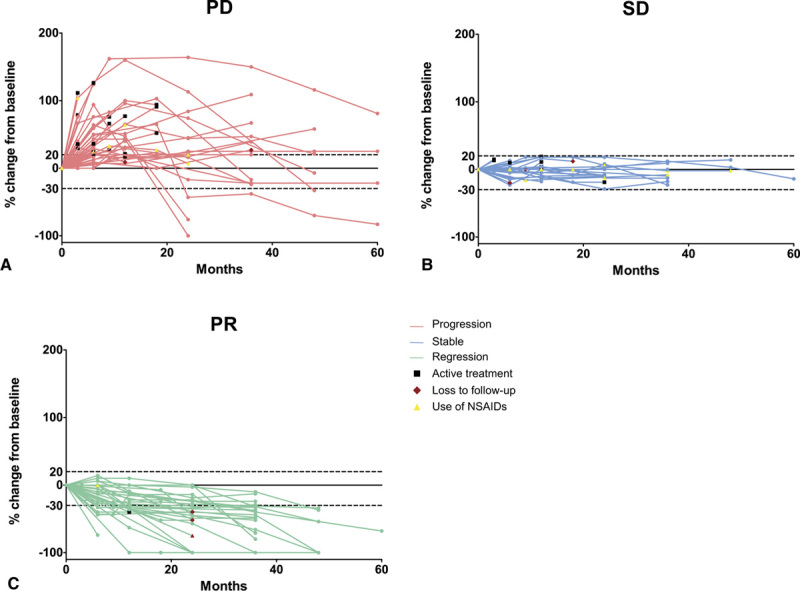
Spider plot of relative change of largest desmoid-type fibromatosis diameter from baseline over time for all evaluable patients (*n* = 104), defined as those with baseline tumor assessments and at least 1 postbaseline assessment. (A) Patients with progressive disease (PD) during follow-up (FU) (*n* = 42);(B) patients with stable disease (SD) during FU (*n* = 33);(C) patients with partial regression (PR) during FU (*n* = 29). Horizontal dashes lines represent ≥20% increase in tumor size compared to baseline (PD according to RECIST) and ≥30% decrease in tumor size according to baseline (PR according to RECIST). FU, follow-up. Pink, PD; Blue, SD;Green, PR;Circle, imaging measurement;Yellow triangle, NSAID use;Red diamond, loss to FU;Black square, start of active treatment.

The natural behavior of DTF tumors of 104 patients was assessed during follow-up. One patient received active treatment within 3 months after inclusion due to symptomatic progression; hence tumor behavior was not monitored. For 9 patients MRI was not available and computed tomography (*n* = 4) or US (*n* = 5) images were used to assess tumor growth. After start of AS, 42 DTF tumors showed initial progression (40%), 33 remained stable (32%), and 29 solely demonstrated partial or CR (28%; Fig. 3).

PFS at 1 year was 69% (95% CI 60–78) and 58% (95% CI 49–69) at 3 years. With a median follow-up of 33.7 months, median time to PFS was not reached (Supplemental Digital Content Fig. 3A).

An increase in tumor size was not observed after a patient demonstrated a decrease in tumor size at ≥3 consecutive imaging examinations (Table 3, http://links.lww.com/SLA/D687).

**Table 3:** Comparison of Patients With Progressive Disease who Continued Active Surveillance Versus Patients With Progressive Disease who Switched to Active Treatment

	PD and Continue Active Surveillance (*n* = 21) *n* (%)	PD and Switch to Active Treatment (*n* = 21) *n* (%)	*P*
Age at time of inclusion (years)			0.533
<40	11 (52%)	13 (62%)	
≥40	10 (48%)	8 (38%)	
Sex			0.292
Male	7 (33%)	4 (19%)	
Female	14 (67%)	17 (81%)	
Tumor size at baseline (cm)			
Median (IQR)	4.0 (3.0–5.8)	5.6 (3.8–8.0)	0.043^*^
<5	15 (71%)	7 (33%)	0.013^**^
≥5	6 (29.6%)	14 (67%)	
*CTNNB1* mutation status (*n* = 40)^1^			0.058
Other^2^	8 (42%)	2 (10%)	
T41A	9 (47%)	15 (71%)	
S45F	2 (11%)	4 (19%)	
Co

Comparative analyses were performed with χ^2^ tests for categorical variables and Mann-Whitney *U* test for continuous variables.

^*^Difference in median tumor size, calculated with Mann-Whitney *U* test.

^**^Difference in tumor size <5 compared to ≥5 cm, calculated with χ^2^ test.

^1^Patients with unknown *CTNNB1* mutation status were not included in the comparative analysis.

^2^Other: S45P, S33L, H36P, Ser33Tyr, or wild-type (WT) mutations.

## DISCUSSION

The GRAFITI trial is a prospective study evaluating patients with non-intraabdominal DTF who underwent AS as initial management. This study shows that two-thirds of the DTF patients undergoing AS do not need an active treatment during follow-up after a median follow-up of 33.7 months. The majority of the DTF tumors remained stable or regressed during follow-up, even after initial progression. Patients with a S45F mutation have a higher risk of starting an active treatment.

Currently, AS is already recommended as upfront approach for the management of DTF.^18^ This recommendation was based on the results of several retrospective studies with different patient cohorts and various follow-up schedules and definitions of AS.^12,14,15,20,23^ In this study, failure of the AS approach was seen in 30% of patients, which is comparable to previous retrospective studies.^24^ More than 50% of these patients needed a change in treatment strategy within the first year after diagnosis. None of the patients of whom follow-up was available started active treatment after year 3. These findings indicate that with an initial AS approach, patients can be reassured that the likelihood of the need to start an active treatment diminishes over time.

Identifying subgroups with risk of failure of AS will help selecting the appropriate treatment strategy and follow-up procedure upfront. Tumor localization, age at diagnosis, *CTNNB1* mutation status, and tumor size are most frequently reported as potential clinicopathological factors associated with recurrence, tumor behavior, or change in treatment strategy in DTF patients.^13,17,19–21,24–27^ In this study, a larger tumor size at baseline (≥5 cm) was associated with a higher risk to start active treatment in the univariable analysis. This finding was also reported in previous retrospective studies,^19,20^ although the predictive value of tumor size was not confirmed by Colombo et al.^13^ It has been reported that the S45F mutation is associated with a higher risk of recurrence in surgically treated DTF patients, suggesting a more aggressive behavior.^26,28^ The influence of *CTNNB1* mutations on change in treatment strategy was not investigated previously. This study showed that the presence of a S45F mutation is an independent predictor for initiation of active treatment. Tumor size was not associated with initiation of active treatment in the multivariable analysis. The latter may be explained by the limited number of patients harboring the S45F mutation, which resulted in wide CIs. In addition, the relatively low number of patients who started active treatment (*n* = 31) may have led to insufficient power to find a significant effect for tumor size on the necessity to start active treatment. Interestingly, the majority of the DTF tumors harboring the T41A mutation were <5 cm and tumors harboring a S45F mutation were significantly larger compared to other mutation types. Timbergen et al^28^ also suggested an association between *CTNNB1* mutation and tumor size based on the results of their meta-analysis. Hence, it could be hypothesized that tumor size at baseline does influence the risk of starting an active treatment after an initial AS approach.

The present study did not assess the predictive value of tumor localization due to the limited numbers, although patients with DTF located at the head and neck and upper extremity experienced more PD and more often needed a switch to active treatment. This is in line with a study by Penel et al,^16^ who found that DTF located at unfavorable locations (head and neck, upper extremity, and chest wall) experienced more PD and more often needed active treatment. A study by Van Houdt et al^19^ showed that upper extremity and chest wall tumors caused more pain, possibly leading to a higher need for active treatments. Further exploration of the predictive value of tumor localization could be of added value.

PD mainly occurred within the first 2 years. One patient developed PD according to RECIST after 3 years; however, her DTF tumor did show a constant increase over time. None of the patients who demonstrated a decrease in tumor size eventually developed or returned to PD. Additionally, patients with PD who started active treatment had significantly larger tumors compared to patients with PD who continued AS, supporting the hypothesis that tumor size does matter. It is interesting to note that in the group of patients with PD who did continue with AS, the majority of the DTF tumors stabilized or even regressed after initial PD.

These findings have important implications for the AS strategy of DTF patients and their follow-up schedules. As PD and initiation of active treatment most likely occur within the first 3 years, DTF patients with an initial AS approach should be monitored for 3 years. However, when a patient shows a decrease in tumor size at ≥3 consecutive imaging examinations, it is unlikely that the DTF tumor will start to grow. Therefore, a more flexible or shorter follow-up schedule can be considered for these patients. If a DTF tumor continues to grow since the start of follow-up, follow-up should be continued to evaluate whether the tumor eventually stabilizes or if there is an indication for active treatment due to increase in symptoms or a high risk of morbidity. After 3 years, the treating physician and patient will make a shared decision how follow-up will be continued, based on tumor behavior, symptom burden, and the patient’s needs. These implications regarding the follow-up strategy must be interpreted with caution for pregnant DTF patients undergoing AS, given the currently limited data available.

The majority of patients in whom active treatment was initiated had PD. However, for most of these patients, it was a combination of PD and an increase in symptoms which necessitated the start of active treatment. Two patients with PD started active treatment due to a pregnancy wish, although it is debatable if this is a strong indication for active treatment. Ten patients with SD or even with regression also received an active treatment because of pain or functional complaints, which was consistent with the study by Van Houdt et al.^19^ Nine patients started with NSAIDs due to pain caused by their DTF tumor and were able to continue AS safely. Adequate pain control as a first step may therefore prevent the need to switch to more aggressive antitumor treatments in DTF patients.^5^

This present study is subject to several limitations. First, the pain score was not well documented in the majority of patients, leading to missing data. Only the presence and progression of symptoms as assessed by the treating physician were reported; severity of symptoms was not scored. Objective symptom scores were therefore not used in the current study. Presence of symptoms may be biased by the potentially different assessment of symptoms by different physicians. However, it can be argued that this subjective method is consistent with current daily practice in determining the treatment strategy for DTF patients. Furthermore, all decisions to start an active treatment were discussed in multidisciplinary meetings and the international guidelines for active treatment were followed to the extent possible.^18^

Second, follow-up of patients who started with active treatment after initial AS was not available in the current study to evaluate the outcomes of these active treatments. However, there is no reason to believe that these outcomes would differ from the retrospective data from previous studies in the Dutch population.^29,30^ Finally, patients underwent for practical reasons both MRI and US examinations during follow-up. In all analyses, tumor behavior was solely based on MRI, as US could not be used as a method of measurement according to the RECIST guidelines,^22^ resulting in large time intervals between RECIST measurements. However, the number of patients experiencing PD, SD, and PR in our study is comparable with previous studies.^14,19^ Furthermore, RECIST may not be the most useful tool to evaluate treatment success in DTF. These criteria assume spherical-shaped tumors and a uniform decrease in size, whereas DTF can display variable shapes with infiltrative growth.^31–33^ Subsequently, tumor size in DTF remains an ambiguous variable which is prone to interobserver variability. Tumor volume or MRI T2 signal intensity, may be better parameters to evaluate radiological response in DTF.^25,33^ In addition to radiological response, health-related quality of life measurements could help to determine treatment efficacy, especially because not all patients with a high symptom burden show PD.^5^ During an AS approach, changes in health-related quality of life scores are a reason to re-evaluate the AS strategy and could help to identify patients who need some form of active treatment.

The small study cohort, although relatively large given the rarity of DTF, limited the analyses of clinicopathological factors associated with start of active treatment. Considering the low incidence of DTF, collaborations are essential. In France and Italy, similar studies (ClinicalTrials.gov identifier NCT01801176 and NCT02547831, respectively) have been conducted to prospectively evaluate AS in DTF patients. Combining the results of these 3 prospective studies will help to further identify subgroups at risk of failure of the AS approach.

In conclusion, this study indicates that after AS, only a minority of DTF patients will need active treatment, minimizing overtreatment and potential morbidity. The majority of DTF patients eventually will develop stable or regressive disease. *CTNNB1* mutation status and tumor size could be used to identify patients with risk of failure of AS. These results may help to tailor the follow-up schedule according to growth behavior and the patient’s needs during follow-up, leading to a more personalized approach.
